# Festina Lente

**Published:** 1882-11

**Authors:** 


### FESTINA LENTE.

Progress is desirable. Real and rapid advance in scientific knowledge is pre-eminently a consummation devoutly to be wished. The improvement and perfection of the means for combatting human ills is worthy of the zealous labor of the ablest and best among the sons of men. Efforts to reclaim outlying territory, to extend the boundaries of usefulness and to widen the borders for the exercise of properly directed energy are praiseworthy, and ought to be cultivated. All inventions and discoveries calculated to render the application of our art more exact are desirable, and should be encouraged. To these propositions, we apprehend, there can be found no dissenting voice. But let us consider. Is there not danger to medical interests and institutions in the unrestrained and eager race for something new which marks the age?

It behooves us seriously to inquire if the temptation to pronounce premature conclusions and to render hasty and inconsiderate opinions is not too strong for successful resistence when the surging of the great crowd of enthusiastic professional workers, impressed by the contagious influence which emulation engenders, is felt all around us; when each individual member is straining every nerve to secure his own preferment and to possess himself of some of the very few prizes within the possible reach of the medical practitioner.

We have been led into this line of thought by observing the prevalence of what appears to us to be a tendency toward superficial and careless reasoning, and a loose and haphazard practice—toward the substitution of glittering generalities for close and critical analysis, and a flippant and frequently expressed distrust in the true and tried therapeutical resources of our art. A moment's reflection will convince any observant physician that we are not alarmed without cause, and, moreover, that the evil indicated assumes many different phases.

An ingenious pharmaceutist, for instance, concocts a new remedy or combines a few old ones; a captivating and suggestive title is invented and applied, profuse advertising is indulged, samples are generously distributed, the preparation is used, amendment follows, a certificate is returned, signed, perhaps, by a prodigious professor; and forthwith all the little men prescribe the new remedy, draw their conclusions, report their cases, sign more certificates, gain an ephemeral notoriety, help to swell the profits of the manufacturer until the new becomes old, then the bubble bursts, and months—it may be years—are required to correct an evil which a heedless haste has pecipitated.

An imaginative pathologist thinks he perceives a new bug or a peculiar cell. His wonderful discovery is promptly heralded. Other men see what he has seen. Without undue formality or unnecessary delay, this "hitherto undescribed" insect is at once decided to be the cause of a certain disease, whereupon the pathology of the familiar disorder is rearranged, the therapeutics is readjusted and made to match the new theory; remarkable cures follow, all goes well, and everything is lovely until the novelty wears away, the charm is broken, the delusion is dispelled.

Examples could be multiplied almost indefinitely and illustrations furnished *ad libitum.* Take vaccination· Humanized lymph has protected us; it served our fathers well; it has saved millions of lives, and yet it seems destined to succumb to the professional and public clamor for something new.

Again, mothers' milk once served for infants' food, or in its unavoidable absence properly prepared cows' milk afforded an acceptable substitute, but now, alas! the innocents are slaughtered—slaughtered with something new; and the newer, the more pretentious, and the more hypocritical the claims of the manufactured product the more popular and acceptable it seems to be.

But we forbear. The danger must surely be apparent to the watchful, and if it is seen they will certainly appreciate the necessity for prudence, steadiness and conservatism at the helm.

It is well to make haste, but let us always remember to make haste slowly.

